# Mapping the respiratory microbiome in intubated children over time

**DOI:** 10.1128/spectrum.00448-25

**Published:** 2025-10-20

**Authors:** Keiko M. Tarquinio, Jennifer Farrell, John J. Varga, Conan Zhao, Elijah Mehlferber, Sam P. Brown

**Affiliations:** 1 Department of Pediatrics, Emory University School of Medicine, Children’s Healthcare of Atlanta 1367, Atlanta, Georgia, USA; 2 School of Biological Sciences, College of Sciences, Georgia Institute of Technology 123387, Atlanta, Georgia, USA; 3 Center for Microbial Dynamics and Infection, Georgia Institute of Technology 1372, Atlanta, Georgia, USA; NHLS Tygerberg/Stellenbosch University, Cape Town, South Africa

**Keywords:** respiratory microbiome, pediatrics, intubation

## Abstract

Children who require airway intubation are almost always treated with empiric antibiotics, pending clinical microbiology results. Positive pathogen results are near inevitable, due to the frequent presence of potential opportunistic pathogens, whether they are causing disease or not. This pattern leads to potential antibiotic over-prescribing, to the detriment of both patients who do not require antibiotics and to antimicrobial stewardship goals. To assess our hypothesis that opportunistic pathogens are more common in children with prior lung disease, we prospectively profiled tracheal aspirate (TA) microbiome samples in children with and without prior lung disease. An IRB approval was obtained, and TAs were collected longitudinally. Samples were analyzed using 16S rDNA sequencing and conventional cultures. Patient demographics and clinical courses were obtained using electronic medical records. Thirteen subjects were included, producing 39 TA samples. Microbiome analysis identified 98 bacterial genera, dominated by *Pseudomonas* and *Streptococcus*. Patient identity was a significant determinant of TA sample variation, indicating a robust individual TA microbiome despite the presence of substantial antibiotic exposures. In contrast, clinical category and time since intubation were not significant predictors, in the context of substantial inter-patient variation. Our results reveal substantial inter-patient variation in TA microbiome structure, limiting our ability to test for significant impacts of clinical category on microbiome structure. Our results provide information for the design of larger-scale studies to evaluate the role of clinical history and specific taxa in governing the interplay between antibiotics and pathogen dynamics in critically ill children.

**IMPORTANCE:**

Clinicians often prescribe empirical antibiotics for critically ill, intubated children with suspected respiratory infections, contributing to antibiotic overuse and challenging antimicrobial stewardship. Our longitudinal tracheal aspirate analysis of cultures and 16S rDNA sequencing revealed significant inter-patient variability, regardless of the primary reason for intubation. We observed both concordance and discrepancies between clinical microbiology and sequencing results—gram-negative organisms aligned well between methods, whereas *Streptococcus* was detected in 34 of 39 samples by 16S rDNA but only once by culture. Our findings emphasize the value of longitudinal airway microbiome analysis in pediatric patients. Given the heterogeneous pathologies and diverse age groups in pediatric intensive care, future large-scale studies should account for antibiotic exposure, commensal bacterial interactions, and clinical conditions that influence microbiome dynamics. Expanding research in this area could improve our understanding of microbial shifts in critically ill children and inform more targeted treatment strategies.

## INTRODUCTION

When children require airway intubation, they are typically started on empirical antibiotics while awaiting culture-dependent microbiological results (1, 2). However, clinical microbiology often returns positive results for colonization due to the polymicrobial nature of the lungs and respiratory tract, where opportunistic pathogens are frequently detected, regardless of their role in active infection (3–5). This pattern contributes to antibiotic over-prescribing, which not only exposes patients to unnecessary treatments but also undermines antimicrobial stewardship efforts (2, 6, 7). Excessive antibiotic use carries significant risks, including increased costs, potential adverse effects such as nephrotoxicity, and the promoting of antibiotic resistance (8). Despite these concerns, clinicians frequently initiate empirical antibiotics based on clinical presentation and laboratory findings, particularly in critically ill children requiring intubation due to suspected respiratory infections (9, 10).

Like any community, lower respiratory microbiomes are shaped by fundamental biogeographic ecological processes, including immigration (micro-aspiration), emigration (cough, sneezing), and differential reproduction and survival (11–14). However, once patients are intubated, the balance of these processes shifts. Aspiration likely increases as the endotracheal tube (ETT) provides a direct conduit from the mouth to the lower respiratory tract, allowing oral and gastric secretions to enter the lungs (15, 16). Additionally, most children require sedation and, in some cases, neuromuscular blockade to ensure the ETT remains safely and comfortably in place during acute respiratory failure (17). These medical interventions compromise natural defense mechanisms and possibly alter host immune responses, reducing the ability to combat infection (18, 19). More recently, the concept of colonization blooming has been described, wherein host factors, such as acute viral infection, medical treatment, and nutrient availability, contribute to biofilm dispersion. This transition to a planktonic state increases bacteria virulence and infection potential (20, 21). Lastly, the broad-spectrum antibiotic use is common in intubated patients in pediatric intensive care units (PICUs) (2, 10, 22). This selective stress against colonized microbiomes alters microbial compositions by favoring species based on their resistance profiles, further shaping the respiratory microbiome (3, 23, 24).

Obtaining samples for lower respiratory tract (LRT) microbiome analysis is challenging due to the risk of sample contamination exit from the LRT. “Gold standard” direct sampling methods (tissue biopsy and bronchoscopy [25]) are limited by their invasive nature and typically are only conducted when there is a direct clinical need. Given the invasive nature of these direct sampling, the majority of LRT microbiome analyses in intubated children rely on profiling tracheally aspirated (TA via ETT) secretions (26, 27). Using TA profiling, Tsitsiklis et al. discovered that the incidental carriage of potentially pathogenic microbes occurred in 68% of children without LRT infections (28). One longitudinal microbiome study concluded that changes over time in microbiome factors, such as bacterial load and dominant taxa, were minimal in ventilator-associated infections (29). However, another study concluded that alpha diversity decreased and risk of infection increased with increasing duration of intubation (30).

In light of this prior work, we sought to longitudinally characterize the TA microbiome of children in three distinct clinical categories based on the primary clinical presentation leading to the intubation: (A) children with primary pulmonary pathologies (including pneumonia, bronchiolitis, pulmonary hemorrhage, and acute respiratory distress syndrome); (B) children with preexisting non-pulmonary organ involvement, or “systemic disease” (e.g. liver dysfunction, cardiac etiology, and sepsis); and (C) children with no previous pulmonary and/or other systemic organ diseases or so-called “previously healthy” but required intubation (e.g., trauma victims with altered mental status without direct lung trauma, muscle weakness without lung infections, or procedural sedation requiring intubation). We hypothesized that children with prior lung damage would suffer higher rates of opportunistic pathogen colonization, due to prior deficits in barrier function.

## RESULTS

### Demographics

**Fig 1:**
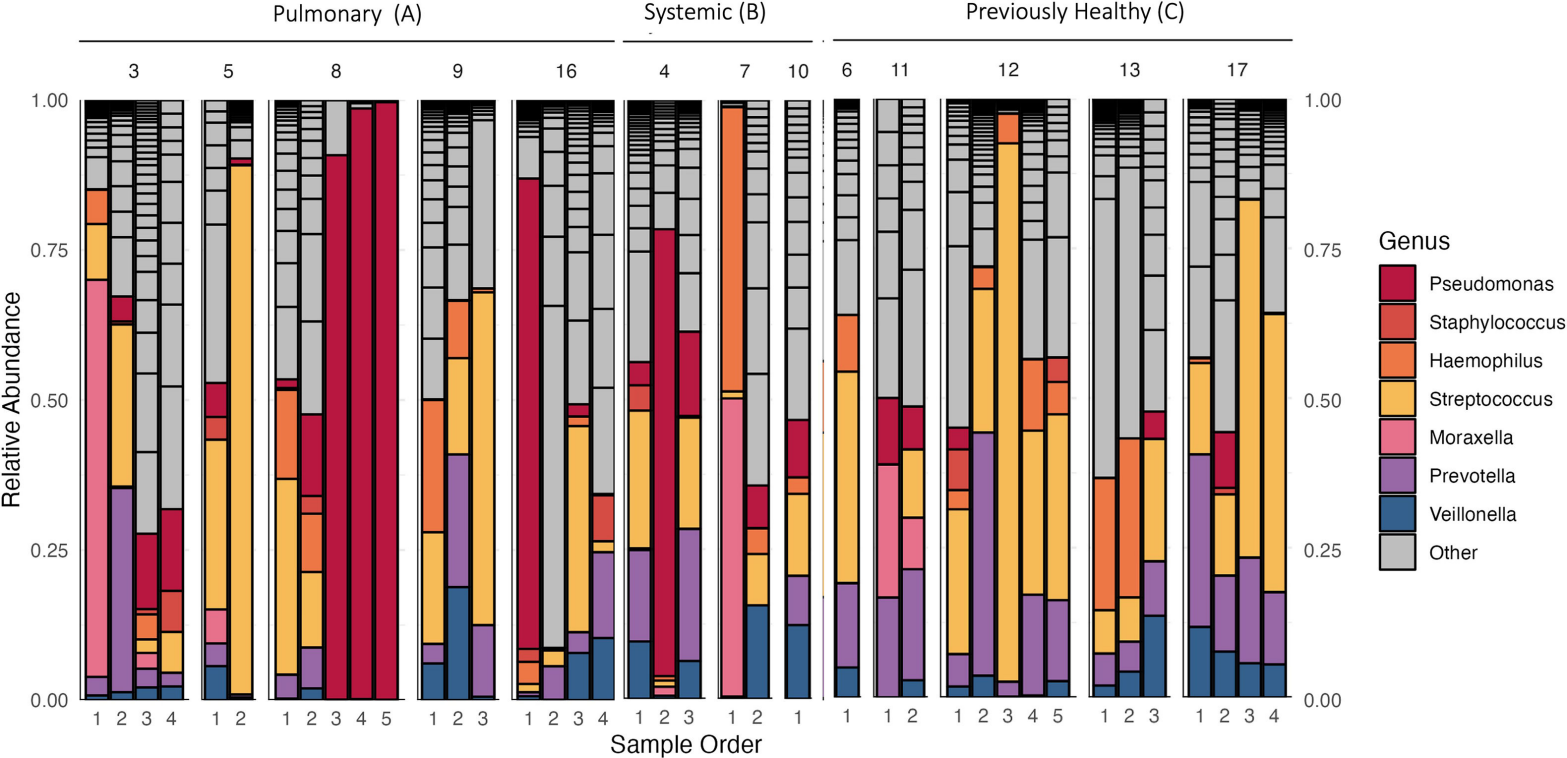
Relative abundance of bacterial genera in each subject. Relative abundances of key taxa (as determined by 16S rDNA sequencing) by patient, grouped by clinical category. The x-axis is based on time and sample collection (1 = 1^st^ sample collection). Taxa shown are the top seven identified taxa that make up 1% or greater of the total bacterial abundance in the whole cohort. “Other” genera are known as non-human pathogens.

A total of 15 subjects were screened, and 13 subjects enrolled who provided 36 TAs (mean of 2.8 TA per subject) over a mean intubation duration of 5 days, yielding 36 conventional cultures and 39 samples for 16S rDNA sequencing (Table 1). Samples with <10^4^ genomes per mL TA were reported as below the detection limit (BDL, Fig. 1). The primary diagnoses leading to intubation were categorized into three groups: the “primary pulmonary” etiology group (category A, *N* = 5, 38%); the non-pulmonary, “systemic disease” group (category B, *N* = 3, 23%); and the “previously healthy” lung group considered as no previous pulmonary or other systemic organ diseases but an acute illness leading to intubation (category C, *N* = 5, 38%) (Table 1). Category A included viral bronchiolitis, necrotizing pneumonia, pulmonary hemorrhage, community-acquired pneumonia, and acute respiratory distress syndrome. All category A patients had acute respiratory illness, including one with chronic lung disease from prematurity. Category B was diagnosed with septic shock, liver disease, and cardiac arrest. One patient with chronic pulmonary illness (controlled asthma) presented with acute liver failure as a primary etiology and was therefore categorized in B. Category C had relatively healthy lungs and was intubated secondary to neuromuscular disease or airway protection for altered mental status or trauma without lung injury (Table 1; Table S1, https://github.com/Cybertarquink/Airway-Microbiome_Supplemental-Materials).

**TABLE 1:** Demographics: subject characteristics^*a*^

Characteristics	*N*
Total enrollment	13
Median age in years (IQR)	1.8 (1.2–10.8)
0–1 (y/o)	7
2–5 (y/o)	0
6–16 (y/o)	6
Male sex (percentage)	9 (69%)
Race	
Black or African-American	7
White	5
Asian	1
Primary diagnosis (reasons for intubation)	N (%)
Category A: Pulmonary (pneumonia, bronchiolitis, pulmonary hemorrhage, acute respiratory distress syndrome)	5 (38)
Category B: Systemic disease (sepsis, liver failure, cardiac arrest)	3 (23)
Category C: Previously healthy (trauma, neuromuscular diseases, procedural intubations)	5 (38)
No other co-morbidity (%)	7 (54)
Other Co-morbidity^*b*^	
Chronic lung disease, asthma	2
Prematurity	2
Airway abnormality (airway malacia, OSA)	3
Congenital heart disease	1
Neurological disease	2
Genetic syndrome (chromosomal abnormality)	1
Total TA collection	36
TA samples (mean per subject)	2.8
Median duration of intubation (days) (IQR)	5.0 (4.0–10.0)
Antibiotic use	N (frequency)
Never^*d*^	1
Always on antibiotics^*c*^	11
Started after 1st sample obtained^*e*^	1
Ceftriaxone	7
Vancomycin	6
Piperacillin/tazobactam	5
Clindamycin	2
Cefazolin	2
Ampicillin, gentamicin, azithromycin, doxycycline, ampicillin/sulbactam^*d*^	1

^*a*^ IQR, interquartile range; OSA, obstructive sleep apnea; TA, tracheal aspirate.

^*b*^ Co-morbidity may manifest more than one condition in the same subject.

^*c*^ Subjects received antibiotics during the intubation period.

^*d*^ “Never”: never exposed to antibiotics.

^*e*^ “Started after 1st sample obtained”: after 1st sample obtained, the antibiotics was initiated on one subject.

All but two patients had more than one sample collection (Fig. 1; Table S2, https://github.com/Cybertarquink/Airway-Microbiome_Supplemental-Materials). The median age of the enrolled subjects was 1.8 years (IQR 1.2–10.8) with a bimodal distribution and male predominance. During the enrollment, we did not have subjects with ages 2–5 years. All but three subjects received antibiotics upon or prior to intubation.

### Culture-dependent analysis

We analyzed 36 cultures using standard hospital procedures for bacteria and fungus. Conventional culture identification was performed in the CHOA clinical microbiology laboratory using routine institutional practices (see the details under the Materials and Methods section).

Among all the cultures we obtained, *Pseudomonas aeruginosa*, *Enterobacter cloacae*, and yeast were most frequently identified, followed by *Moraxella catarrhalis*, *Staphylococcus aureus* (including methicillin-resistant *S. aureus*), and *Haemophilus influenzae*. About half of the initial cultures showed no growth (46%), while two cultures were positive for *M. catarrhalis* (Table S2, https://github.com/Cybertarquink/Airway-Microbiome_Supplemental-Materials). Persistent *P. aeruginosa* and *E. cloacae* growth over time was observed in one subject (patient P8) with neurological co-morbidity (Table S1, https://github.com/Cybertarquink/Airway-Microbiome_Supplemental-Materials).

### 16S rDNA profiling of TA microbiome

16S rDNA amplicon sequencing yielded 635,941.2 mapped reads across 39 samples and 98 genera. Figure 1 illustrates the compositional structure of the microbiome samples, organized by the patient (panel) and sampling time point (x-axis). Across all 39 samples, the most prevalent taxa (presence defined by >1% relative abundance) were *Streptococcus* (present in 34/39 samples), *Prevotella* (30/39), *Veillonella* (25/39), *Haemophilus* (24/39), and *Pseudomonas* (21/39) among collected samples. Individual patients showed a variety of dominant taxa (taxa with over 50% relative abundance in at least one time point), including *Moraxella* (patients P3 and P7), *Pseudomonas* (patients P4, P8, and P17), and *Streptococcus* (patients P5, P9, P12, and P16), indicating substantial inter-patient variability in microbiome structure. A linear mixed effects model reveals that alpha diversity differences across disease type and time are not significant (Fig. S1, https://github.com/Cybertarquink/Airway-Microbiome_Supplemental-Materials).

**Fig 2:**
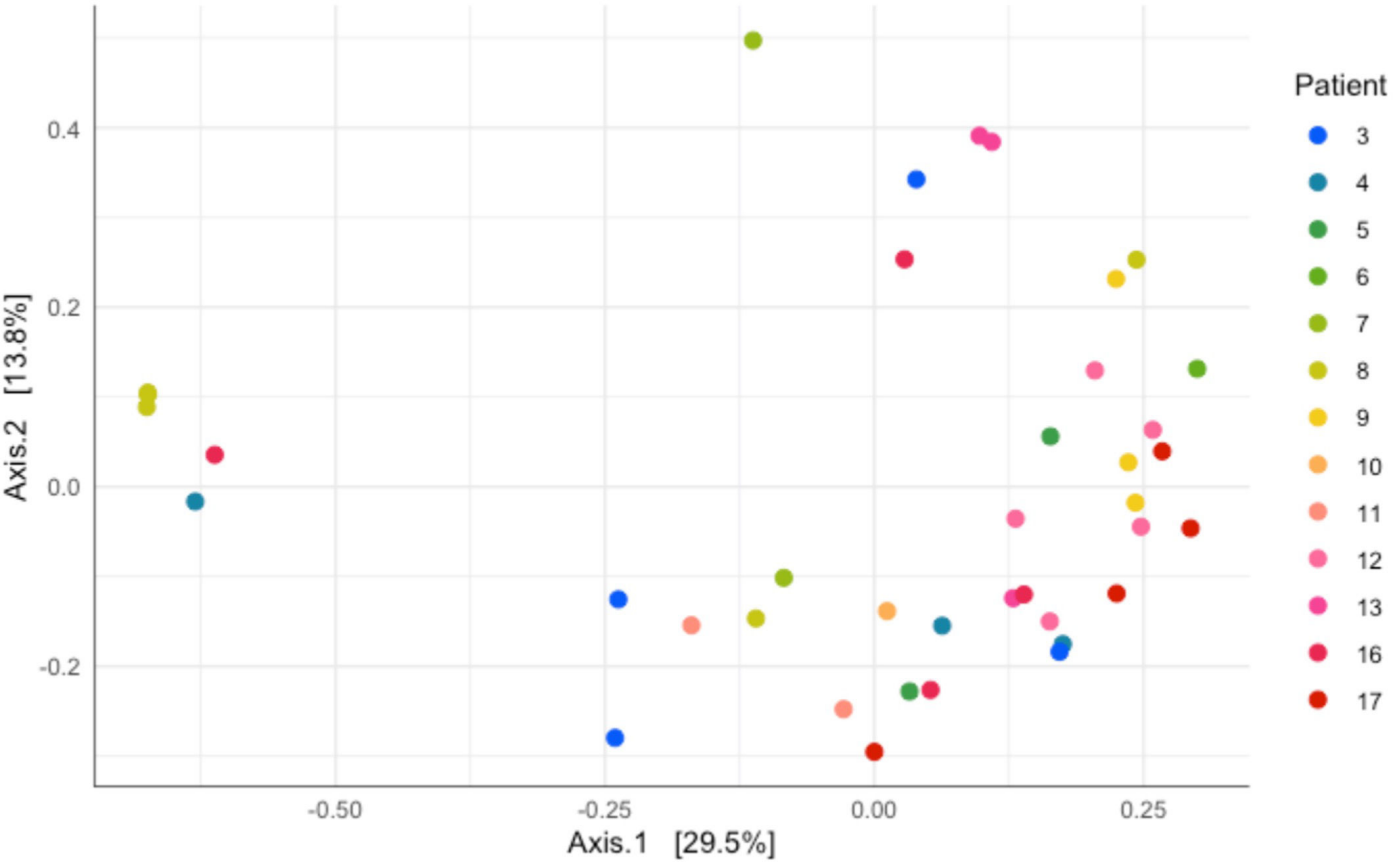
Principal coordinate analysis (PCoA) ordination of microbiome community from each patient. Each dot represents a time point, and each color represents an individual patient. Relative abundance was log-ratio transformed. PERMANOVA showed significant clustering by the individual patient (F = 1.751, *P* = 0.003), but not by clinical category (F = 1.527, *P* = 0.106) or duration of intubation (F = 1.926, *P* = 0.079).

**Fig 3:**
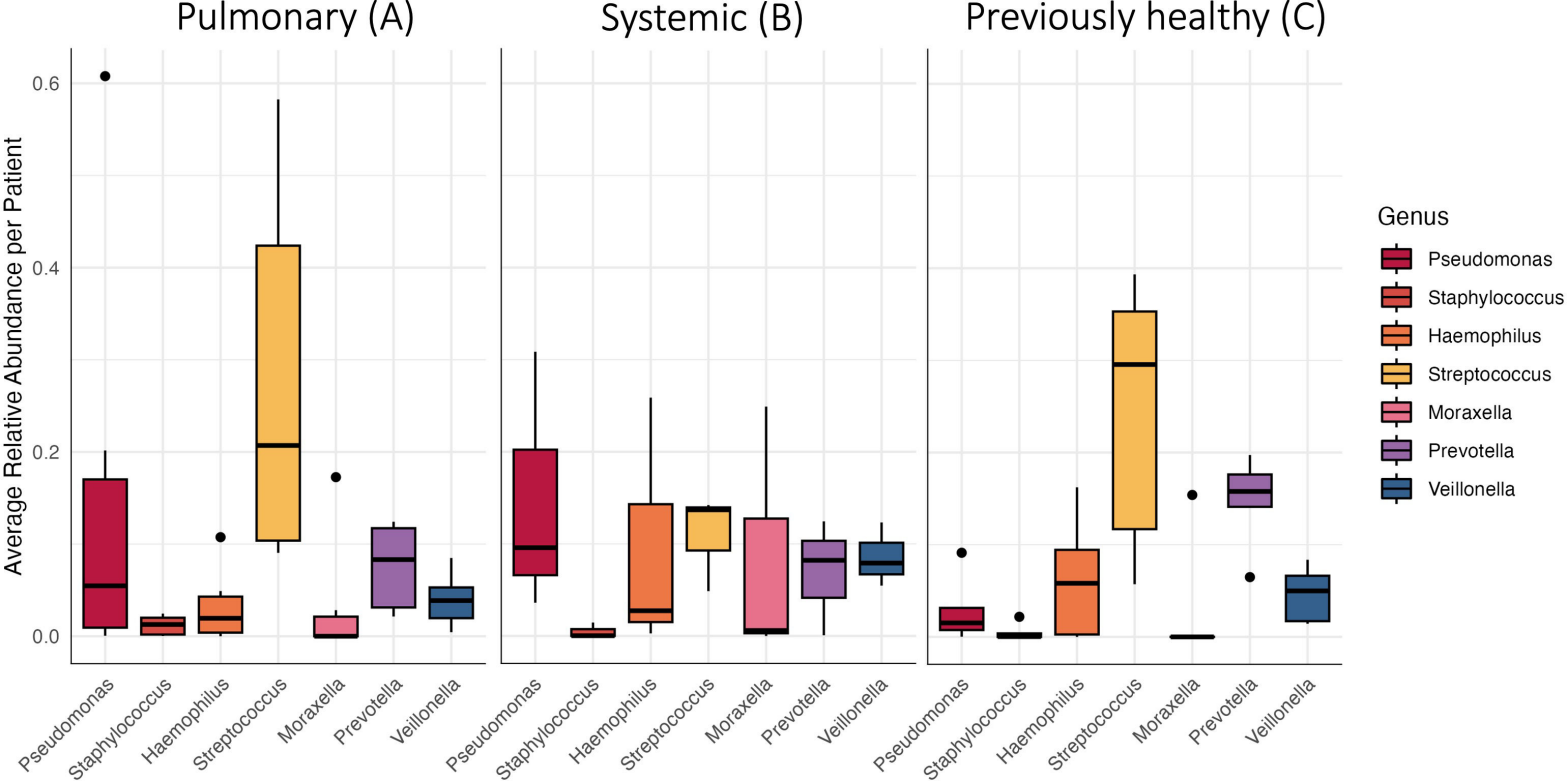
The abundant genera by patient categories. Relative abundance of taxa grouped by clinical categories (**A** primary pulmonary etiology, **B** systemic diseases, **C** previously healthy lungs). Black dots represent individual data points.

**Fig 4:**
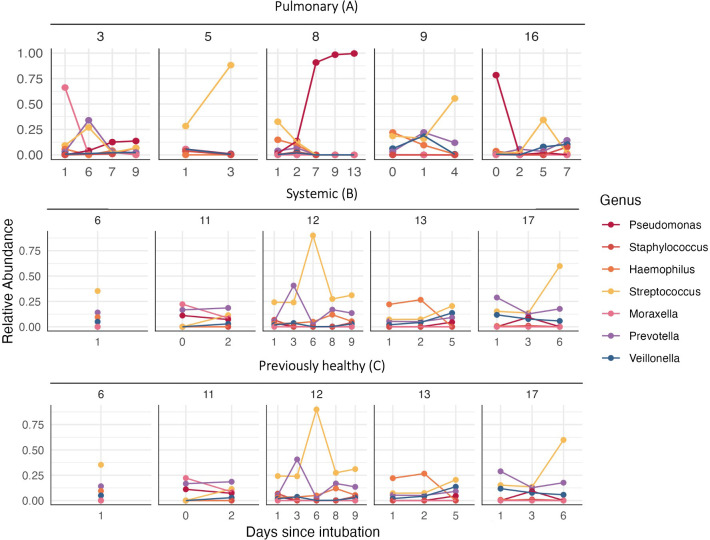
Longitudinal trend of relative abundance of key taxa for each patient in three categories. There was no specific pattern of taxon distribution by the duration of intubation or by clinical category.

To begin to explore this variation, we next re-plotted the data in Fig. 2 as a two-dimensional ordination plot of microbiome clustering using principal coordinate analysis (PCoA). The PCoA with each sample colored showed significant clustering by the individual patient (PERMANOVA, F = 1.751, *P* = 0.003). However, the clustering was not significant by clinical category (F = 1.527, *P* = 0.106) or duration of intubation (F = 1.926, *P* = 0.079). Given the absence of community-scale differences by clinical category, we next proceed cautiously with our motivating hypothesis of increased opportunistic pathogens in children with prior lung damage. Our 16S rDNA profiling reveals several taxa that potentially contain defined respiratory pathogen species (*Moraxella*, *Pseudomonas*, *Neisseria*, *Haemophilus*, and *Streptococcus*) (Fig. 3). We note that some of these genera are also home to a multitude of purely commensal species (in particular, *Streptococcus*). To limit loss of statistical power, we therefore focus our statistical test on *Pseudomonas*, as this was among our most common genera and we have higher confidence that the reads in this genus will relate to the established opportunistic pathogen species, *Pseudomonas aeruginosa*. Our statistical analysis showed a significant difference in *P. aeruginosa* relative abundance among all diagnostic categories (Kruskal-Wallis chi-squared = 7.4531, df = 2, *P* = 0.02408), but no difference in abundance in individual category comparisons between primary pulmonary and previously healthy (category A and C, Dunn’s test, *P* = 0.0723), systemic and previously healthy (category B and C, Dunn’s test, *P* = 0.0638), and primary pulmonary and systemic (category A and B, Dunn’s test, *P* = 1.000) (Fig. 3). Patients 3 and 16 showed a relatively high abundance of *Moraxella* and *Pseudomonas* on the first sample despite receiving antibiotics (Fig. 4).

## DISCUSSION

This preliminary surveillance study highlights substantial variability in tracheal aspirate (TA) microbiomes from intubated children, with most variation attributable to individual patient differences (Fig. 1 and 2). Due to the observed inter-patient heterogeneity and small sample size, our study lacked the statistical power to test our primary hypothesis regarding a distinct, pathogen-enriched TA microbiome in children with prior lung damage (Fig. 3). Principal coordinate analysis (PCoA) ordination and PERMANOVA revealed no clear microbiome patterns associated with primary diagnosis or with duration of intubation. These findings aligned with previous studies on ventilator-associated infections (29, 30).

Our study assesses only bacterial abundances by 16S sequencing; thus, the presence or abundance of eukaryotes (e.g. yeasts) or viruses was not determined by molecular methods. Recently, bacterial colonization “bloom” has been described in the context of acute viral infections, where preexisting biofilm-associated colonizers disperse into a planktonic state, increasing their pathogenic potential (20, 21). Unlike biofilms, which exhibit significant antibiotic resistance even at high concentrations, planktonic bacteria are generally more susceptible to antimicrobial treatment (31).

We acknowledge that our study could not assess the interaction between bacterial infections and potential virus co-infections. However, yeast presence was detected in some bacterial cultures from our study samples. Increasing evidence suggests that bacterial colonizers in the nasal or upper respiratory tract can transition into true pathogens under specific conditions (24, 32, 33). This knowledge gap is particularly relevant, given the widespread use of broad-spectrum antibiotics in our cohort, which may have promoted yeast overgrowth by suppressing the commensal microbial community (34). Future studies incorporating broader profiling of eukaryotes and viruses alongside bacterial analysis could bring new insights into the temporal evolution of airway microbiota from health to disease (20, 32). Our study also highlights both concordance and discrepancies between clinical microbiology reporting and 16S rDNA results. While gram-negative organisms showed general agreement between culture-based identification and 16S rDNA profiling (with both methods detecting the same organisms in five patients), several discrepancies were observed. For example, clinical cultures detected *Streptococcus* only from Patient 05 on the day of intubation (Table S2, https://github.com/Cybertarquink/Airway-Microbiome_Supplemental-Materials), whereas 16S rDNA sequencing identified *Streptococcus* in 34 out of 39 samples. This discrepancy likely stems from clinical microbiology focus on culturing pathogenic *streptococci*, while 16S rRNA sequencing captures a broad diversity of commensal *Streptococcus* species (35, 36).

Similar findings have been reported in studies comparing culture-based and sequencing-based diagnostics in dental caries and lower respiratory infections, suggesting that the transition from colonization to disease involves complex interactions between microbial communities and environmental factors (36, 37). Consequently, associations between colonization and disease should be interpreted with caution. A broader disconnect was observed in 15 samples that were reported as “no growth” by clinical microbiology, yet showed diverse bacterial microbiomes by 16S rRNA sequencing. This likely reflects the ability of sequencing-based approaches to detect commensal organisms that fall outside the scope of routine clinical culture methods. While many microbiome studies provide only cross-sectional snapshots of microbial communities in health and disease, our study contributes to understanding longitudinal microbiome changes within the same cohort (38).

We acknowledge several limitations in our study beyond its bacteriocentric focus. First, the absence of a true “healthy” cohort presents a challenge, as ethical concerns preclude the intubation of healthy children solely for research purposes. As an alternative, we assumed that category C (previously healthy children) would best approximate a non-diseased lung environment. However, this assumption remains theoretical and was not directly validated in our study. Second, the small sample size was further fragmented across diagnostic categories, limiting our ability to detect significant differences between groups. Additionally, multiple clinical factors, such as oxygen content and delivery, bacterial nutrient availability, temperature fluctuations, and host immune responses, were not adjusted due to the limited sample size. These variables likely influence microbiome ecology, particularly bacterial growth dynamics (11–13). Further studies with larger cohorts and improved statistical control of clinical variables may provide deeper insights into the impact of preexisting lung disease on the microbial dynamics of lung infection susceptibility.

### Conclusions

Our results reveal substantial inter-patient variability in TA microbiome composition, overshadowing differences between clinical categories. Given this variability, we were unable to detect significant microbiome among groups. By characterizing longitudinal inter-patient variation, our study establishes a benchmark for designing future larger scale studies aimed at identifying microbiome signature associated with specific clinical conditions in intubated pediatric patients.

## MATERIALS AND METHODS

### Study design, setting, and participants

We conducted a single-center prospective cohort study in a 36-bed, quaternary center PICU in the U.S.A. with an average annual admission of 2,800. We enrolled subjects who met the inclusion criteria.

Inclusion criteria were (1) intubated children who are likely to be mechanically ventilated >48 hours and (2) age 0 to 21 years old. Exclusion criteria were (1) likely extubated within 24–48 hours and (2) contraindication or higher risks for suction via ETT such as post-operative airway reconstructions. All demographic characteristics, medical history, and details of the acute illness were collected during hospitalization, and all culture results were obtained from the electronic medical charts.

### Specimen collection

Initial TA was collected within 24 hours of intubation time, followed by collections on the next Monday, Wednesday, and Friday, continuing until either extubation or 14 days had passed—whichever came first. Collection of TA was performed per ICU routine practice using a new sterile, appropriately sized in-line suction catheter (Halyard, Alpharetta, GA) which was attached to a Lukens specimen trap (Cardinal Health, Dublin, OH), striving for the first suctioning ideally each morning. Every sample collection was performed using a freshly changed in-line catheter to minimize the contamination. One milliliter of sterile normal saline was also suctioned through the catheter into the Lukens trap, and the remainder of normal saline was saved for negative control of 16S rDNA sequencing. All samples were immediately transferred to the clinical microbiology laboratory at the Children’s Healthcare of Atlanta (CHOA), aliquoted in cryovial tubes, and stored at −80°C frozen until DNA prep was performed at the Brown laboratory at the Georgia Institute of Technology (GA Tech).

Conventional culture identification was performed in the CHOA clinical microbiology laboratory using routine institutional practices. Briefly, the TA was plated to blood agar, chocolate agar, MacConkey agar, CNA, and anaerobic culture media, streaking in four quadrants. Then, plates were incubated at 34–37°C in CO2, and anaerobic culture was incubated at 34–37°C anaerobically. The smear was prepared for gram stain from purulent portions of the specimen. The specimen was rejected if showing greater than 10 squamous epithelial cells (SECs) per low power field (10× objective). Probable pathogens were identified, including *Enterobacteriaceae*, *Pseudomonas aeruginosa,* and other non-fermenting gram-negative rods, fungi to include yeast, *Streptococcus pneumoniae*, *Streptococcus pyogenes*, *Staphylococcus aureus*, *Haemophilus influenzae*, and *Moraxella catarrhalis*. The plates were incubated for 18–24 hours and observed at 24 and 48 hours. The isolates were subcultured as needed onto blood agar, chocolate agar, or MacConkey agar for isolation from mixed cultures to perform susceptibility testing when appropriate. Cultures were interpreted by clinical microbiologists at the CHOA laboratory by examining the quantity and types of bacteria presence.

### 16S rDNA sequencing and compositional analysis

Genomic DNA was isolated using the Qiagen Power Soil Pro kit (Qiagen Inc., Germantown, MD, USA). Library preparation and sequencing were performed by Swift Bioscience (Ann Arbor, MI, USA). Briefly, libraries were generated using the Swift Normalase Amplicon Panel targeting all nine variable regions of the bacterial 16S rDNA sequence and subjected to Illumina sequencing. The resulting raw sequencing reads were processed using the Swift Biosciences 16S-SNAPP pipeline (https://github.com/swiftbiosciences/16S-SNAPP) using default parameters, with the exception of the read cutoffs (230 bp for forward sequences and 180 bp for reverse sequences). The processing pipeline removes primers using Cutadapt, then quality filters and denoises reads before joining the paired ends (39). Chimeras were removed using DADA2, yielding amplicon sequence variants (ASVs) (40). A unique sequence set was generated with VSEARCH and used to query a custom 16S database of high-identity matches from RDP 11.5 (www.rdp.cme.msu.edu)(41). Read classification was performed against this database to generate OTU counts. Data were then converted into a PhyloSeq object (42), and read counts were converted to relative abundance. Prior to analyzing beta diversity, a rarefaction curve was produced (43), indicating an ideal depth of 7,000 reads per sample, which would lead to the removal of four low-read samples. Analysis of differences in beta diversity using an Adonis PERMANOVA [VEGAN] (43) was performed on data that was rarefied to 7,000 reads and on data that was rarefied to 1,800 reads (to keep all samples). Results were quantitatively identical, and so a rarefaction depth of 1,800 reads per sample is presented in the paper. Data were rarefied to a depth of 1,800 reads for visualization of the beta diversity using a PCoA, while all other figures present un-rarefied relative abundance. A linear mixed effects model with disease type and time as fixed effects, and patient ID as a random effect, was constructed to determine the impact of these variables on community diversity.

Our data (a bioproject number: PRJNA 1289159) was uploaded.
